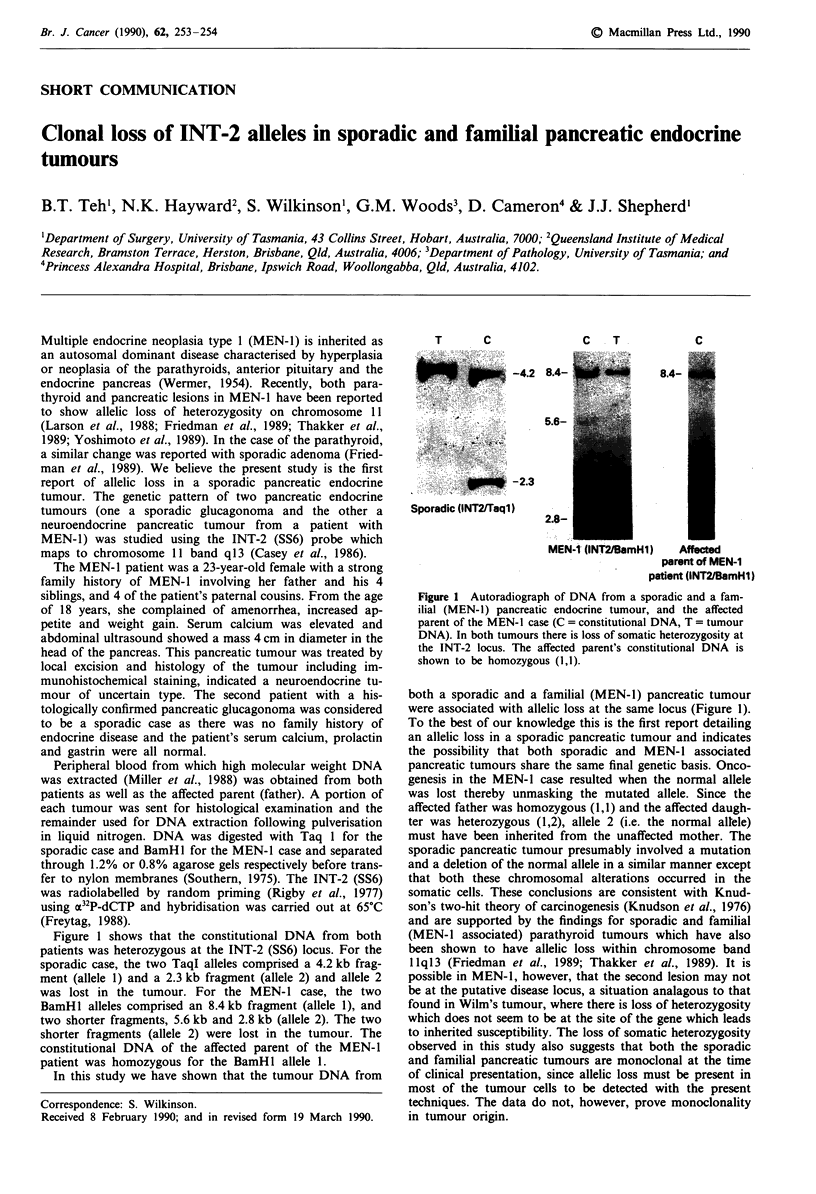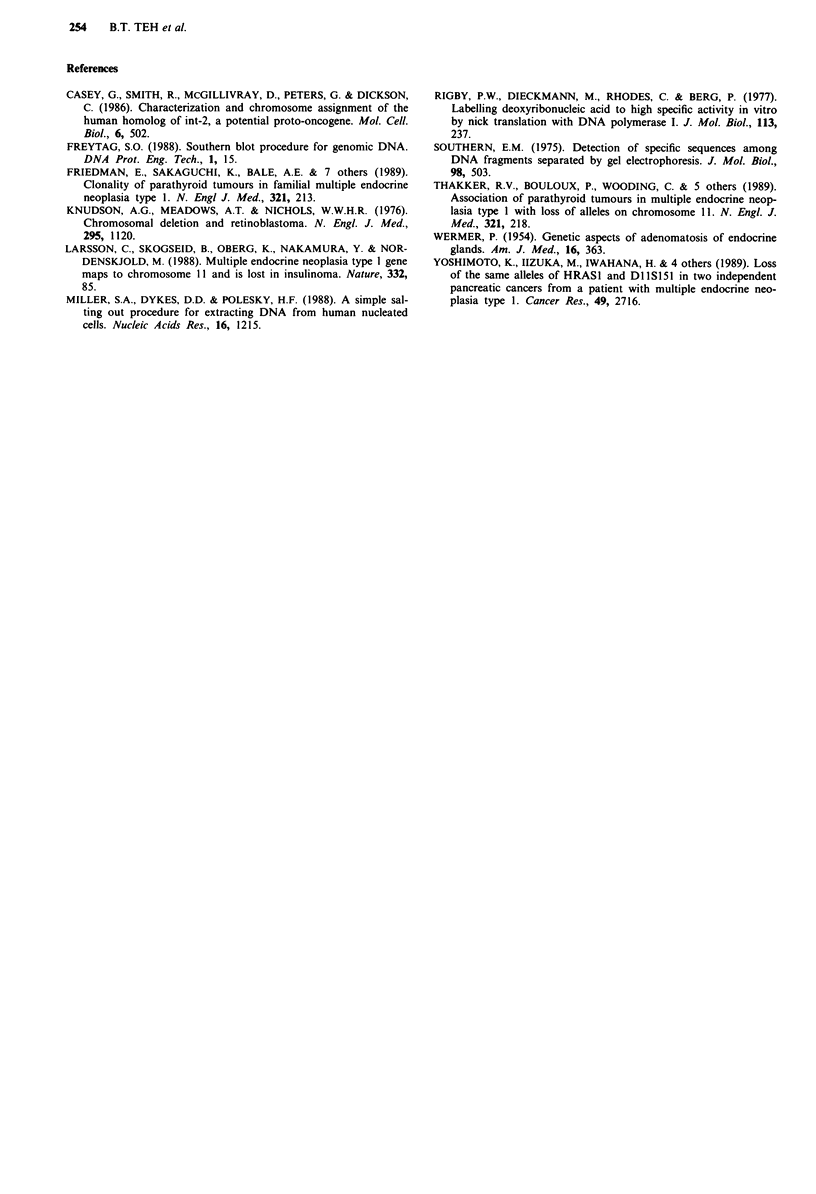# Clonal loss of INT-2 alleles in sporadic and familial pancreatic endocrine tumours.

**DOI:** 10.1038/bjc.1990.270

**Published:** 1990-08

**Authors:** B. T. Teh, N. K. Hayward, S. Wilkinson, G. M. Woods, D. Cameron, J. J. Shepherd

**Affiliations:** Department of Surgery, University of Tasmania, Hobart, Australia.

## Abstract

**Images:**


Br. J. Cancer (1990), 62, 253-254 © Macmillan Press Ltd., 1990

SHORT COMMUNICATION

### Clonal loss of INT-2 alleles in sporadic and familial pancreatic endocrine tumours

B.T. Teh[1], N.K. Hayward[2], S. Wilkinson[1], G.M. Woods[3], D. Cameron[4] & J.J. Shepherd[1]

[1]*Department of Surgery, University of Tasmania, 43 Collins Street, Hobart, Australia, 7000;* [2]*Queensland Institute of Medical Research, Bramston Terrace, Herston, Brisbane, Qld, Australia, 4006;* [3]*Department of Pathology, University of Tasmania; and* [4]*Princess Alexandra Hospital, Brisbane, Ipswich Road, Woollongabba, Qld, Australia, 4102.*

Multiple endocrine neoplasia type 1 (MEN-1) is inherited as an autosomal dominant disease characterised by hyperplasia or neoplasia of the parathyroids, anterior pituitary and the endocrine pancreas (Wermer, 1954). Recently, both parathyroid and pancreatic lesions in MEN-1 have been reported to show allelic loss of heterozygosity on chromosome 11 (Larson *et al.*, 1988; Friedman *et al.*, 1989; Thakker *et al.*, 1989; Yoshimoto *et al.*, 1989). In the case of the parathyroid, a similar change was reported with sporadic adenoma (Friedman *et al.*, 1989). We believe the present study is the first report of allelic loss in a sporadic pancreatic endocrine tumour. The genetic pattern of two pancreatic endocrine tumours (one a sporadic glucagonoma and the other a neuroendocrine pancreatic tumour from a patient with MEN-1) was studied using the INT-2 (SS6) probe which maps to chromosome 11 band q13 (Casey *et al.*, 1986).

The MEN-1 patient was a 23-year-old female with a strong family history of MEN-1 involving her father and his 4 siblings, and 4 of the patient's paternal cousins. From the age of 18 years, she complained of amenorrhea, increased appetite and weight gain. Serum calcium was elevated and abdominal ultrasound showed a mass 4 cm in diameter in the head of the pancreas. This pancreatic tumour was treated by local excision and histology of the tumour including immunohistochemical staining, indicated a neuroendocrine tumour of uncertain type. The second patient with a histologically confirmed pancreatic glucagonoma was considered to be a sporadic case as there was no family history of endocrine disease and the patient's serum calcium, prolactin and gastrin were all normal.

Peripheral blood from which high molecular weight DNA was extracted (Miller *et al.*, 1988) was obtained from both patients as well as the affected parent (father). A portion of each tumour was sent for histological examination and the remainder used for DNA extraction following pulverisation in liquid nitrogen. DNA was digested with Taq 1 for the sporadic case and BamH1 for the MEN-1 case and separated through 1.2% or 0.8% agarose gels respectively before transfer to nylon membranes (Southern, 1975). The INT-2 (SS6) was radiolabelled by random priming (Rigby *et al.*, 1977) using $\alpha^{32}$P-dCTP and hybridisation was carried out at 65°C (Freytag, 1988).

Figure 1 shows that the constitutional DNA from both patients was heterozygous at the INT-2 (SS6) locus. For the sporadic case, the two TaqI alleles comprised a 4.2 kb fragment (allele 1) and a 2.3 kb fragment (allele 2) and allele 2 was lost in the tumour. For the MEN-1 case, the two BamH1 alleles comprised an 8.4 kb fragment (allele 1), and two shorter fragments, 5.6 kb and 2.8 kb (allele 2). The two shorter fragments (allele 2) were lost in the tumour. The constitutional DNA of the affected parent of the MEN-1 patient was homozygous for the BamH1 allele 1.

In this study we have shown that the tumour DNA from

**Figure 1** Autoradiograph of DNA from a sporadic and a familial (MEN-1) pancreatic endocrine tumour, and the affected parent of the MEN-1 case (C = constitutional DNA, T = tumour DNA). In both tumours there is loss of somatic heterozygosity at the INT-2 locus. The affected parent's constitutional DNA is shown to be homozygous (1,1).

both a sporadic and a familial (MEN-1) pancreatic tumour were associated with allelic loss at the same locus (Figure 1). To the best of our knowledge this is the first report detailing an allelic loss in a sporadic pancreatic tumour and indicates the possibility that both sporadic and MEN-1 associated pancreatic tumours share the same final genetic basis. Oncogenesis in the MEN-1 case resulted when the normal allele was lost thereby unmasking the mutated allele. Since the affected father was homozygous (1,1) and the affected daughter was heterozygous (1,2), allele 2 (i.e. the normal allele) must have been inherited from the unaffected mother. The sporadic pancreatic tumour presumably involved a mutation and a deletion of the normal allele in a similar manner except that both these chromosomal alterations occurred in the somatic cells. These conclusions are consistent with Knudson's two-hit theory of carcinogenesis (Knudson *et al.*, 1976) and are supported by the findings for sporadic and familial (MEN-1 associated) parathyroid tumours which have also been shown to have allelic loss within chromosome band 11q13 (Friedman *et al.*, 1989; Thakker *et al.*, 1989). It is possible in MEN-1, however, that the second lesion may not be at the putative disease locus, a situation analagous to that found in Wilm's tumour, where there is loss of heterozygosity which does not seem to be at the site of the gene which leads to inherited susceptibility. The loss of somatic heterozygosity observed in this study also suggests that both the sporadic and familial pancreatic tumours are monoclonal at the time of clinical presentation, since allelic loss must be present in most of the tumour cells to be detected with the present techniques. The data do not, however, prove monoclonality in tumour origin.

Correspondence: S. Wilkinson.
Received 8 February 1990; and in revised form 19 March 1990.

### References

CASEY, G., SMITH, R., MCGILLIVRAY, D., PETERS, G. & DICKSON, C. (1986). Characterization and chromosome assignment of the human homolog of int-2, a potential proto-oncogene. *Mol. Cell. Biol.*, **6**, 502.

FREYTAG, S.O. (1988). Southern blot procedure for genomic DNA. *DNA Prot. Eng. Tech.*, **1**, 15.

FRIEDMAN, E., SAKAGUCHI, K., BALE, A.E. & 7 others (1989). Clonality of parathyroid tumours in familial multiple endocrine neoplasia type 1. *N. Engl J. Med.*, **321**, 213.

KNUDSON, A.G., MEADOWS, A.T. & NICHOLS, W.W.H.R. (1976). Chromosomal deletion and retinoblastoma. *N. Engl. J. Med.*, **295**, 1120.

LARSSON, C., SKOGSEID, B., OBERG, K., NAKAMURA, Y. & NORDENSKJOLD, M. (1988). Multiple endocrine neoplasia type 1 gene maps to chromosome 11 and is lost in insulinoma. *Nature*, **332**, 85.

MILLER, S.A., DYKES, D.D. & POLESKY, H.F. (1988). A simple salting out procedure for extracting DNA from human nucleated cells. *Nucleic Acids Res.*, **16**, 1215.

RIGBY, P.W., DIECKMANN, M., RHODES, C. & BERG, P. (1977). Labelling deoxyribonucleic acid to high specific activity in vitro by nick translation with DNA polymerase I. *J. Mol. Biol.*, **113**, 237.

SOUTHERN, E.M. (1975). Detection of specific sequences among DNA fragments separated by gel electrophoresis. *J. Mol. Biol.*, **98**, 503.

THAKKER, R.V., BOULOUX, P., WOODING, C. & 5 others (1989). Association of parathyroid tumours in multiple endocrine neoplasia type 1 with loss of alleles on chromosome 11. *N. Engl. J. Med.*, **321**, 218.

WERMER, P. (1954). Genetic aspects of adenomatosis of endocrine glands. *Am. J. Med.*, **16**, 363.

YOSHIMOTO, K., IIZUKA, M., IWAHANA, H. & 4 others (1989). Loss of the same alleles of HRAS1 and D11S151 in two independent pancreatic cancers from a patient with multiple endocrine neoplasia type 1. *Cancer Res.*, **49**, 2716.